# Supplementary material for: Application of Multigene Panels Testing for Hereditary Cancer Syndromes
Source: Biology (Basel). 2022 Oct 5;11(10):1461. doi: 10.3390/biology11101461 (PMC9598138; doi:10.3390/biology11101461)
Supplement: Supplementary file 1 [file biology-11-01461-s001.zip › Table S2.pdf]

Table S2. List of databases used for variant interpretation

CGD, HGNC, Ensembl , RefSeq, CIViC genes, dbSNP, GERP, ClinVar, IARC TP53 germline, IARC TP53 somatic, ICGC germline, ICGC somatic, Kaviar, gnomAD Exomes, gnomAD Genomes, DANN scores, CIViC mutations, HPO, UniProt variants, UniProt domains, GWAS Catalog, GHR, DGV, DECIPHER, ClinVar, ExAC CNVs, PanelApp, MONDO, PMKB, DOMINO, GDC, Bravo, MitoMap, SIFT, SIFT4G, PROVEAN, LRT, MutationTaster, MutationAssessor, FATHMM, fitCons, MetaSVM and MetaLR, Eigen & Eigen PC, M-CAP, REVEL, MutPred, MVP, MPC, PrimateAI, deogen2, ALoFT, phyloP, phastCons, bStatistic, GTEx, UniProt Genes, BioCarta Pathway, ConsensusPathDB, KEGG pathway, GDI, LoFTool, Essential genes, GHIS, RVIS, HIPred, P(HI) Score, P(rec) Score, GNF/Atlas Expressions, Gene Ontology, AACT, DGIdb, COSMIC, Polyphen-2, PharmGKB, CGD, HGNC, Ensembl, RefSeq, UniGene, CIViC genes, dbSNP, GERP, ClinVar, IARC TP53 germline, IARC TP53 somatic, ICGC germline, ICGC somatic, Kaviar, gnomAD Exomes, gnomAD Genomes, DANN scores, CIViC mutations, HPO, UniProt variants, UniProt domains, GWAS Catalog, GHR, DGV, DECIPHER, ClinVar, ExAC CNVs, PanelApp, MONDO, PMKB, DOMINO, GDC, Bravo, MitoMap, SIFT, SIFT4G, PROVEAN, LRT, MutationTaster, MutationAssessor, FATHMM, fitCons, MetaSVM and MetaLR, Eigen & Eigen PC, M-CAP, REVEL, MutPred, MVP, MPC, PrimateAI, deogen2, ALoFT, phyloP, phastCons, bStatistic, GTEx, UniProt Genes, BioCarta Pathway, ConsensusPathDB, KEGG pathway, GDI, LoFTool, Essential genes, GHIS, RVIS, HIPred, P(HI) Score, P(rec) Score, GNF/Atlas Expressions, Gene Ontology
