# Supplementary material for: Application of Multigene Panels Testing for Hereditary Cancer Syndromes
Source: Biology (Basel). 2022 Oct 5;11(10):1461. doi: 10.3390/biology11101461 (PMC9598138; doi:10.3390/biology11101461)
Supplement: Supplementary file 1 [file biology-11-01461-s001.zip › Table S1.pdf]

Table S1. List of genes studied

MTHFR, SDHB, MUTYH, RAD54L, PTGFR, DPYD, GSTM1, CHRNB2, FDPS, SDHC, FCGR2A, FCGR3A, F5, SERPINC1, CYB5R1, EPHX1, PARP1, EPCAM, MSH2, MSH6, FANCL, GGCX, BUB1, PROC, POLD1, BARD1, ATIC, UGT1A9, UGT1A4, UGT1A1, FANCD2, VHL, MLH1, BAP1, PROS1, UMPS, ATR, ADD1, PDGFRA, KIT, UGT2B15, UGT2B10, COQ2, FAM175A, ABCG2, EGF, FASTKD3, MTRR, HTR1A, HMGCR, APC, RAD50, CTNNA1, SPINK1, ADRB2, TNF, FANCE, FKBP5, KIF6, CYB5R4, OPRM1, SOD2, LPA, PMS2, CRHR2, EGFR, ASL, SEMA3C, CYP3A5, CYP3A4, RINT1, CFTR, CALU, PRSS1, XRCC2, NAT1, NAT2, PPP2R2A, RPS20, NBN, CDKN2A, FANCG, SLC28A3, FANCC, PTGS1, ASS1, RET, SFTPA1, BMPR1A, PTEN, CYP2C19, CYP2C9, CYP2C8, NT5C2, TCF7L2, BUB3, HRAS, CYB5R2, FANCF, F2, GSTP1, MRE11A, ATM, C11orf65, SDHD, H2AFX, GRIK4, CHEK1, GNB3, LRP6, RECQL, CDK4, YEATS4, POLE, BRCA2, RB1, FANCM, RAD51B, MLH3, DICER1, AKT1, TP53BP1, CHRNA3, FANCI, POLG, BLM, NTHL1, SLX4, PALB2, VKORC1, CETP, CDH1, HAS3, FANCA, RPA1, GP1BA, TP53, SLC47A2, NF1, RAD51D, CDK12, ERBB2, BRCA1, NAGS, CRHR1, HOXB13, DLX4, RAD51C, PPM1D, BRIP1, ACE, RHBDF2, TYMS, RBBP8, SMAD4, NEDD4L, MC4R, STK11, LDLR, CYP4F2, BABAM1, CYP2A6, CYP2B6, APOE, POLD1, ITPA, TXNRD2, COMT, ADORA2A, CHEK2, CYP2D6, CYB5R3, FANCB, OTC, G6PD
